# Instrument Nomenclature with Reference to Instrumentation

**Published:** 1898-03

**Authors:** G. V. Black

**Affiliations:** Chicago


### COMMUNICATIONS.

### Instrument Nomenclature with Reference to Instrumentation.

BY G. V. BLACK, M.D., D.D.S., SC.D., CHICAGO.

Read before the National School of Dental Technics, Annual Meeting of 1897.

### INTRODUCTION.

The want of some recognized scheme of nomenclature and classification of dental operating instruments that will individualize the instruments of the several orders and classes is a great bar to progress in teaching instrmentation. This is apparently severely felt by all who labor for exactness in their operations, or endeavor to express the manner of their performance and to speak of the instruments used. The teacher has no means of telling his pupils just what particular instrument he would use in performing a specific act in excavating a given cavity or preparing a margin. The only means of individualizing excavators and pluggers, the instruments which present the most important variations has been the manufacturer's number. These numbers have not been used by the profession, except occasionally for ordering instruments from catalogues. An extended study of these numbers develops the following facts: When an instrument is designed that is thought to be a good pattern the manufacturer assigns it a number. The pattern is preserved as a guide to the reproduction of the form, and each instrument produced is stamped with its number. In this way hundreds of instruments are numbered without system. Finally, to rid himself of the multitude of instruments and numbers, the manufacturer collates those forms that sell best and throws out the rest. He then renumbers those retained, and enters them in a new catalogue. In this way instruments of similar pattern are for a time brought nearer together in the numbering. But the process of adding new numbers begins again and soon it becomes another confused mass. There has never been any system of numbering that indicated either the form or use of the individual instrument. It has been purely arbitrary. For this reason, the numbers have not been used by teachers, except for the one purpose of making lists from which the students should do their buying.

A dentist needs some means of indicating precisely the forms of the instruments used in the operations he endeavors to describe, and especially is this badly needed in school work. So long as the teacher has no means of accurately designating the particular instrument he employs, so long will his manipulative teaching be vague and uncertain. The pupil will be unable to know just what is meant, and any description of the manner of using individual instruments will be confusing and without force.

Not only this, but the confusion gives rise to an unnecessarily great multiplicity of instrument forms. We being unable to teach manipulation, the student fails to learn the range of valuable instrument forms and the powers and capabilities of instruments, and is therefore continually seeking new forms, hoping to find that which will serve him better. This being done without accurate appreciation of mechanical laws, the vast majority of efforts are failures, which tend only to further confuse the manufacturer's numbers.

It should be regarded as a truism that the really capable instruments are strictly limited in present modes of operating by definite laws of usefulness that no man can put aside, and that the valuable forms continue in our cases year after year.

Special instruments for special purposes there are, and many of them fitted only for individual idiosyncracies in operating.

Radically new instruments of value in the line of excavators and pluggers come forward now-a-days only as modes of operating are so changed as to require new forms. This kind of growth will continue. That which is needed now in the line of excavators and pluggers is a strict classification of the useful forms and the development of a scheme of nomenclature for the individual designation of each form. The presentation of such a scheme to the pupil will place before his mind the range of possible forms from the mechanical standpoint, and will enable him to know quickly what has been produced, and in a much fuller sense than heretofore to know what can be produced.

This failure to appreciate instrument forms and the special usefulness of each form gives rise to great confusion in operative procedures. The disagreement as to method among dentists is unnecessarily great, and when such a degree of confusion exists only a very few of the methods can be the best. We should be able to teach methods in our schools. Can we do so without the ability to designate accurately the means of carrying out the method? The carpenter would not buy an auger that had not been made to a definite formula. The carpenter's boy would be laughed at, if, when sent far a quarter-inch auger, he should bring a seven-eighths. Can we not appreciate the forms and sizes of our instruments as definitely?

What our students need in the beginning of school work is a close drill in the appreciation of the forms of cutting instruments and pluggers, such as will enable them to discover the peculiarities of each with exactness, as to width, length and inclination of blades, and the proportions of the several parts. Also they should be instructed as to the possible variation of useful forms. Directly coupled with this, the student should be taught to record the forms for future reference, and, it would be well for him to acquire the skill to reproduce them from the record. If this be coupled with a careful drill in the uses and capabilities of instruments, an impression will be made on the mind, and a skill acquired by the hand that will be a great aid in the development of manipulative ability.

It is my object to develop the details of a scheme of instrument nomenclature and clasisfication applicable to cutting instruments and pluggers, by the use of which the teacher can reach exactness in teaching instrumentation. This will be attained by first defining and arranging in an orderly way the words of instrument nomenclature that have been developed in the ordinary speech of the profession, by use of which groups of instruments may be definitely known, and then arranging a simple system of formulæ by which individual instruments of each group may be accurately designated.

It can not be expected that this proposed scheme for the classification and study of instruments will be of special benefit to dentists now in practice. That is not its object. It is intended for school work only; but may in time spread to the general profession through the students who go out from our schools.

Another object of the scheme is to limit the number of forms of instruments employed, and to adopt a classified list for school work that shall be sufficient for all schools and not be cumbersome to any. This can be done by selecting a sufficient classified list to be used in teaching, and from which each school may select the particular instruments the students will be required to have. This particular feature of the scheme will be more fully developed later. Or, instead of selecting a sufficient classified list for all schools to select from, each school may select its own classified list in accordance with a specified set of rules for the arrangement of instrument sets. A plan for doing this will be developed later, by which any classified set of instruments will be perfectly comprehended in the minutest detail from the written statement by any one familiar with the scheme of classification, and who has attained a working knowledge of any single set of instruments so classified. Also, such instrument sets may be accurately made by any skillful instrument maker without other guide than the written formulæ and the rules that will be developed in this paper.

This paper will be in two parts. The first part will consider and arrange the nomenclature heretofore developed, and the second part will be devoted to the consideration of formula names and the formation of instrument sets.

### PART FIRST.

### INSTRUMENT NOMENCLATURE.

Furnishing the Basis for School instruction.

In the developement of any system of nomenclature, the basis should be the names that have arisen in the common speech of the profession. These names have a meaning, and, if we gain an understanding of this meaning, we will be able to classify the names in accordance with it, and in so doing present an orderly nomenclature. In doing this it is often necessary to choose between two or more names that have been applied to the same thing and occasionally to separate two items that have been called by the same name. In this way the uncertain nomenclature in vogue, developed at random in the first instance, is rendered orderly and definite. This is readily done in instrument nomenclature, and without the introduction of any considerable number of new terms.

### NAMES OF PARTS OF INSTRUMENTS

*Cutting Instruments, or Excavators.*—Each excavator is composed of a shaft which is used as a handle, a shank and a blade. Usually in excavators the shaft is perfectly straight and without variation in size. The *shank* begins with the first turned part and connects the shaft with the *blade* or working point. It usually tapers from its connection with the shaft to where the blade begins.

The *blade* is the part bearing a cutting-edge. It may be said to begin at the angle which terminates the shank—the last one, if there be more than one angle—and ends in a cutting-edge.

*Pluggers* have no cutting-edges and therefore no blades as "A blade is the leaf-like portion of an instrument bearing the cutting-edge." The shank of pluggers, therefore, extends to the working point, though they may have similar angles to the excavators. (We should have a specific name for that portion of the plugger corresponding with the blade of the excavator.)

### CLASSIFICATION OF NAMES OF OPERATING INSTRUMENTS.

Existing names of operating instruments may be divided into order names, sub-order names, class names and sub-class names (4).

An *Order name* is one designating such instruments as are used for a purpose so similar that groups have received a name indicating the purpose of their use, or answers to the question, "What for?"

The well-defined order names are *excavators*, *pluggers*, *separators*, *scalers*, *finishing instruments* and *accessories*.

A *Sub-order name* is one designating the locality, position or manner of use, in such a way as to distinguish certain instruments from other members of the order, or answers the question "Where or how used."

A *Sub-order name* is often attached as a prefix to the order name, as *hand* plugger, *mallet* plugger, *push* scaler, *pull* scaler, etc. *Enamel Trimmer* is a sub-order of excavators. *Burs* belong both to excavators and finishing instruments as sub-orders, as *cavity* bur, *finishing* bur. The word *Bur* is properly a class-name —they have no order name.

A *Class name* is applied to a group of the members of an order and describes the point or immediate working part, as *hatchet* or *hoe*, descriptive of the blades of excavators, or the working point of pluggers, as *convex* plugger, *serrated* plugger, *smooth* plugger, etc.

A *Sub-class name* describes the angles and curves of the shank leading to the working point or blade, as *bayonet* plugger, *spiral* plugger, *contra angle* hatchet excavator.

In the common speech of the profession, these names have been habitually compounded. Sub-order names are prefixed to order names, as in mallet plugger, hand plugger, etc. Class-names are prefixed to order names, as in *hatchet* excavator, *spoon* excavator, *hoe* excavator, etc. Also sub-class names may be prefixed to either order or class names, or all these joined, as in *contra angle hatchet excavator* or *bayonet plugger*.

In all these compoundings, the order name is last, indicating the use or purpose—the sub-order name prefixed, indicating how or where, while the class name is descriptive of the forms of the working point, and the sub-class name the form of the angles and curves of the shank leading to the point. It should be noted particularly that these terms are applied to groups of operating instruments. They specify the kind of instrument but do not individualize the instruments of the group. These may vary indefinitely in the widths, lengths and angles of blades. For these differences we will propose other terms.

RIGHTS AND LEFTS.

There is a distinct division in operating instruments, known as *Rights and Lefts.* Among excavators we have two forms of rights and lefts. The *beveled* rights and lefts and the *lateral cutting* rights and lefts, or, true double plane instruments. The beveled rights and lefts are hatchet forms made rights and lefts simply by the form of the bevel of the cutting-edge. Most of the hatchet forms have bi-beveled edges, i. e., the edge is formed by grinding equally from the two flat sides of the blade. The beveled rights and lefts are formed by making two hatchet forms alike, and then grinding the bevel all from one side on the one, and all from the other side of the blade on the other. The result is a pair of instruments, the one suitable for shaving down the buccal wall of cavity, and the other suitable for shaving down the lingual wall. The cutting-edges are upon opposite sides of the blades, making them rights and lefts. These are used mostly for cutting enamel in opening cavities, but may also be used very effectively in cutting dentin. Any of the hatchet excavators may be made in pairs and converted into beveled rights and lefts, but the general adoption of this, while producing excellent instruments, multiplies the number of instruments in the operating case to such a degree as to cause confusion. For this reason the formation of beveled rights and lefts should be very strictly limited to enamel instruments, or to special instruments for heavy cutting.

LATERAL CUTTING RIGHTS AND LEFTS.

*True Double-Plane Instruments.*—The double-plane, or intersecting plane rights and lefts are a totally different class of instruments, and are designed for lateral cutting, while the other forms, single-plane in struments, are for direct cutting. If any of the single-plane instruments be laid upon a table or any plane surface, in a certain position, it will readily be seen that all of the angles and curves, no matter how many, are in a single plane. If it is held before the eye, in a certain position, the instrument appears straight—such instruments are suited for direct cutting.

If we carefully examine the rights and lefts known as spoons or rapid excavators, it will be noted that each has an angle or curve that is not in the same plane with the principle angle or curve, but in a plane that intersects the plane of this principle angle at right angles. These we will call *double-plane* instruments, they differ essentially from the single-plane instruments in that they are specially suited for lateral cutting. They are always made in pairs. They are first formed similarly to the hatchet excavators, but after the blade is formed the blade of one is curved to the right and the blade of the other is curved to the left. This important division of cutting instruments is confined mostly to what has become known as spoons. They are suited to scooping out masses of carious material. They are not of much value for cutting hard material. This form of rights and lefts is also used occasionally in pluggers,

DEFINITIONS OF CLASS NAMES.

A *Class name* is one that describes the immediate working point of the instrument.

CLASS NAMES OF EXCAVATORS.

*Hatchet.*—The shank has one or more angles or curves, the last length forming the blade, the edge of which is in the plane of the angle or angles.

*Hoe.*—The shank has one or more angles, the last length forming the blade, the edge of which is in a plane intersecting at right angles the plane of the angle or angles.

*Spoon.*—These are always made in pairs. They are first made in the form of hatchets and then the blade of the one is curved to the right and the blade of the other is curved to the left, then the cutting-edge is ground to a semi-circle. This curve of the blade is in a plane that intersects the plane of the principle angle or angles at right angles, making the instruments true rights and lefts.

*Discoids.*—(Disc-like, circular.) The blade is circular in form, having a cutting-edge extending around the whole periphery, except that portion by which it is joined to the shank. This circular blade is placed at more or less of an angle with the shaft.

Formerly this form was called a spoon, several forms being grouped under that name. Discoid blades are sométimes seen on double plane instruments of various forms.

*Cleoids.*—(Claw-like, in the form of a claw.) Sharp pointed blades in the form of a claw, with cutting-edges on two sides of the blade.

*Chisels.*—Straight blades with cutting-edge formed by beveling from one side. The blade is usually straight with the shaft, but may be slightly curved.

*Binangle Chisel.*—A chisel blade placed at a slight angle with the shaft in the hoe form. They are contra-angled.

Rotary cutting instruments will not be included in this list.

SUB-CLASS NAMES.

A *Sub class* name is one applied to and descriptive of the angles and curves of the shank of an instrument which leads to the blade or working point.

*Mon-Angle.*—An instrument having one angle only leading to the working point as in pluggers, or forming the blades as in excavators. Mon-angles form a large majority of excavators. In the greater angles only the shorter blades can be successfully used as mon-angles, for the reason that when the blade is long its inclinatian carries its working point laterally so far from the central line of the shaft as to render the instrument liable to turn in the hand when the edge is forcibly applied. This renders the instrument unsteady and ineffective. To remedy this defect, all cutting instruments, in which the angle and length of blades will carry the cutting-edge more than three millimeters from the line of the central axis of the shaft, should be contra-angled.

*Contra-Angle.*—The shank of the instrument is first bent backward (from the direction of the cutting-edge), and nearer the cutting-edge another bend is made forward—this length forming the blade, the object being to form a long blade, the edge of which will be near the central line of the shaft.

*Binangle Contra-Angle.*—A contra-angle formed by two angles as described under contra-angle.

*Triple Angle Contra-Angle.*— In an instrument of the angle of 12 centigrades or less (about 45 degrees) the binangle contra-angle will bring the cutting-edge sufficiently near the central line of the shaft, and at the same time carry the shank sufficiently out of the way to permit the use of the full length of the blade; but in instruments of a greater angle, a binangle would not do this, therefore a triple angle contra angle must be made; this is done by first bending the shank backward as in the binangle contra angle and then forming another angle which will bring the remainder of the shank parallel with the shaft; then passing forward a space of more or less length as may be required, another bend is made forward by which the blade is formed. In this way the cutting edge of a long blade is brought sufficiently near the central line of the shaft for effective work, and the shank carried sufficiently out of the way to permit the full use of the length of the blade.

Long blades that require contra-angling are mostly for use in places where a long reach of blade is necessary.

There is a number of other sub-class names that have been applied to excavators, but as none of them will be used they will be passed by for the present. Also, there is a number of sub-class names applied to plugger points, as cork screw, cow's horn, bayonet, etc., but as we shall not fully consider pluggers in this paper, they will also be passed.

Curves occur among the rights and lefts or double plane instruments for which no distinctive names have been developed. Those forms which I designate as spoons have a curve beginning at about one-third the length of the blade and gradually increasing to the cutting-edge. Another form often seen, but which now seems to be in less favor, is what I should term the hoe spoon. This blade is straight like that of a hatchet until near the cutting-edge, when it is bent laterally at an angle, and the cutting edge rounded as in the spoons. These are in pairs, as the spoons, and are true double-plane instruments.

Other forms that have been used are almost endless, many of them without names, and very generally have disappeared under the law of unfitness for the purposes intended.

RULES FOR CONTRA ANGLING.—RECAPITULATION.

1st. All blades, the angle and length of which will bring the cutting edge more than three millimeters from the central line of the shaft, should be contra-angled.

2nd. All instruments with angles of 12 centigrades or less, when requiring contra-angles should be binangle contra-angles.

3rd. All instruments with angles of more than 12 centigrades, when requiring contra-angles should be triple-angle contra-angles.

4th. When the contra-angle is used the cutting-edge of the instrument should be brought within two millimeters of the central line of the shaft, or better, when the contra-angle is used the working-edge, should be brought just so near the central line of shaft that when the instrument is laid edge downward upon a plane surface the edge should just touch, but not actually rest upon the surface.

